# Biochemical and transcriptomic analyses of drought stress responses of LY1306 tobacco strain

**DOI:** 10.1038/s41598-017-17045-2

**Published:** 2017-12-12

**Authors:** Huijuan Yang, Li Zhao, Shimin Zhao, Jing Wang, Hongzhi Shi

**Affiliations:** 1 Tobacco College of Henan Agricultural University, No. 95, Wenhua Road, Zhengzhou, Henan Province 450002 P.R. China; 2 Luoyang Tobacco Company, No. 246, Kaiyuan Road, Luoyang, Henan Province 471000 P.R. China

## Abstract

This study aimed to investigate drought resistance of the LY1306 tobacco strain. Seedlings of tobacco strains LY1306, ZhongYan 100 (ZY100) and Hong Hua Da Jin Yuan (HHDJY) were treated with polyethylene glycol (PEG)-6000 to induce osmotic stress. As validation, water-deficit-induced drought treatments, including mild drought (MD; watering 1.5 L/week) and severe drought (SD, without watering) were carried out. Changes in cell morphology, leaf water potential, antioxidant enzyme activity, as well as contents of malondialdehyde (MDA) and proline were determined for each treatment. Transcriptome sequencing was performed for the seedlings treated with 15% PEG-6000. No obvious changes were observed in morphology of LY1306 and ZY100 under osmotic or drought stress; whereas, visible wilting was observed in HHDJY. Superoxide dismutase and peroxidase activities of LY1036 and ZY100 under osmotic stress were significantly higher than those of HHDJY. Under SD, the MDA content of LY1306 was significantly lower and the proline content of LY1306 was significantly higher than that of HHDJY. Differential genes between LY1306, ZY100 and HHDJY were enriched in functions about alpha-linolenic acid, and arginine and proline metabolisms. LY1306 could increase its antioxidant enzyme activities and proline accumulation in response to drought stress, probably by regulating drought resistance-related pathways and genes.

## Introduction

Drought is one of the most common environmental stresses and is commonly defined as a period without significant rainfall^1^. Drought severely constrains plant growth and productivity, which can threaten agroforestry and lead to environmental deterioration^2^, affecting both elongation and expansion growth at the initial phases of plant establishment^3,4^. Further, drought has adverse effects on plant metabolic processes, including nutrient uptake, stomatal movement and production of photosynthetic assimilates, which ultimately results in crop losses^1,5,6^.

Tobacco (*Nicotiana tabacum*), an agriculturally important Solanaceae crop, is one of the most studied plants as a biological model system^7^. Importantly, it is a valuable economic crop and is the most widely grown non-food crop worldwide^8^. Tobacco originates in the tropics under conditions of good rainfall and requires ample water for growth during development. Most tobacco crops entering the world trade are produced in the temperate and tropical regions^9^. According to a Food and Agriculture Organization report, in 2003, China was one of the leading countries growing tobacco^10^. However, currently, drought stress has become a main limiting factor for the production of tobacco in China, particularly in northern China. Therefore, breeding of drought-resistant tobacco varieties is an urgent requirement.

LY1306 is a newly bred tobacco strain obtained through eight years of hybridisation (2005–2012). Considerable field trials (2012–2015) suggest that this strain has stable genetic traits, good yield and quality and high stress and viral disease resistances. However, the underlying physiological and molecular mechanisms have not been investigated.

At the molecular level, most events involved in adaptation probably result from alterations in gene expression^11^. Numerous studies have applied the transcriptomic approach to investigate the drought responses in plants^12,13^, which have provided substantial contributions to our understanding of the molecular mechanisms underlying drought resistance. In the present study, we investigated the drought resistance mechanisms of the LY1306 tobacco strain using biochemical and transcriptomic approaches by comparing with another two tobacco varieties, ZhongYan 100 (ZY100) and Hong Hua Da Jin Yuan (HHDJY). ZY100 is a flue-cured tobacco variety developed by crossing the female parent tobacco strain 9201 and the male parent variety NC82, which presents good adaptability and drought resistanceis, and is resistant to multiple diseases^14^. HHDJY is selected and bred from the variant of Da Jin Yuan, which is superior in quality, but is sensitive to drought^15^. Our data may provide important insight into understanding the drought resistance mechanisms of the LY1306 tobacco strain.

## Results

### Effect of drought stress on morphological changes in LY1306

**Figure 1:**
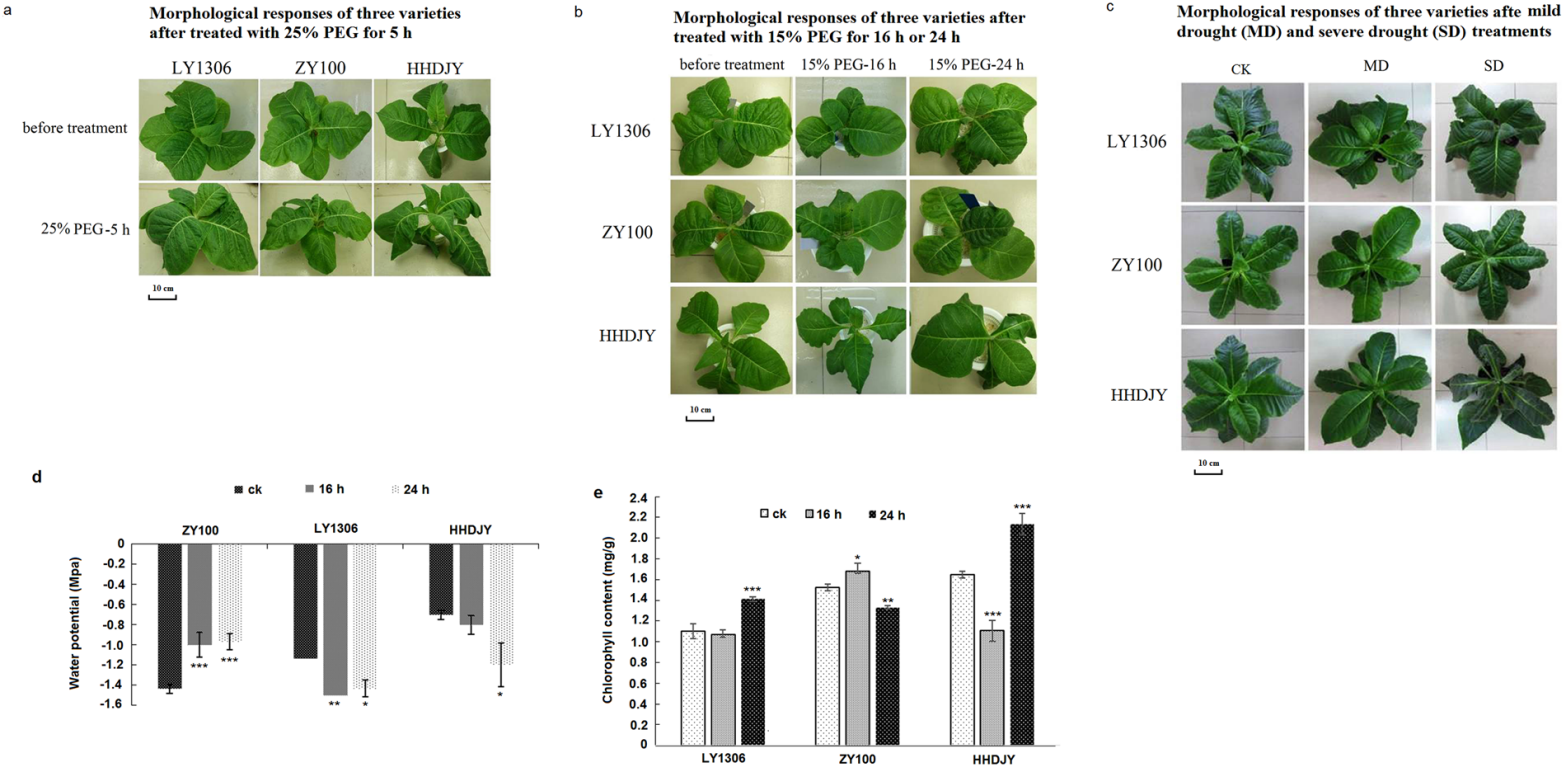
The morphological responses of LY1306, ZhongYan 100 (ZY100) and Hong Hua Da Jin Yuan (HHDJY) tobacco strains under drought stress. (**a**) Seedlings treated with 25% PEG for 5 h; (**b**) Seedlings treated with 15% PEG for 16 h or 24 h; (**c**) Seedlings subjected to mild drought (MD) and severe drought (SD) treatments. (**d**) Changes of leaf water potential in three vatieties after 15% PEG treatment for 0, 16 and 24 h. (**e**) Changes of chlorophyll content in three vatieties after 15% PEG treatment for 0, 16 and 24 h. Results are expressed as the mean ± standard error from three replication experiments. *P < 0.05, **P < 0.01 and ***P < 0.001 compared with the corresponding control groups.

Under normal growth conditions, the growth of LY1306 was similar to that of control strains (ZY100 and HHDJY). After being treated with 25% PEG-6000 for 5 h, the leaves of HHDJY showed visible wilting, whereas those of LY1306 and ZY100 remained normal (Fig. 1a). Moreover, after treating seedlings with 15% PEG-6000 for 16 h, slight wilting was observed in HHDJY, whereas no changes appeared in LY1306. After continuous osmotic stress (15% PEG-6000) for 24 h, there was still no obvious morphological change in LY1306. However, HHDJY exhibited severe wilting (Fig. 1b). In addition to PEG-6000-induced osmotic stress, we induced drought by withholding water supply. Similar to the findings described above, LY1306 exhibited better drought resistance than HHDJY. LY1306 seedlings in the mild drought (MD) group retained their leaf morphology, whereas those in the severe drought (SD) group showed only slight colour changes (Fig. 1c). For HHDJY, SD caused severe wilting and the leaves turned deep green.

In accordance with the findings above, water potential measurement showed that water potential of HHDJY did not change obviously after 16 h of 15% PEG-6000 treatment but decreased significantly after 24 h of treatment (P < 0.05), which suggested that HHDJY failed to respond to osmotic stress quickly. On the contrary, LY1306 decreased leaf water potential after 16 h of 15% PEG-6000 treatment (P < 0.01). Besides, the water potential at 16 h was similar to that at 24 h (P > 0.05). For ZY100, its water potential significantly increased after 16 and 24 h of PEG-6000 treatment (P < 0.001), suggesting that ZY100 may have different drought resistance mechanism with LY1306 (Fig. 1d). Furthermore, the chlorophyll content of HHDJY was significantly higher than that in LY1306 and ZY100 after 24 h of 15% PEG-6000 treatment (P < 0.001) (Fig. 1e), which might explain the phenomenon that the leaves of HHDJY turned deep green in SD group.

### Effect of PEG-6000 on antioxidant enzyme activity of LY1306

**Figure 2:**
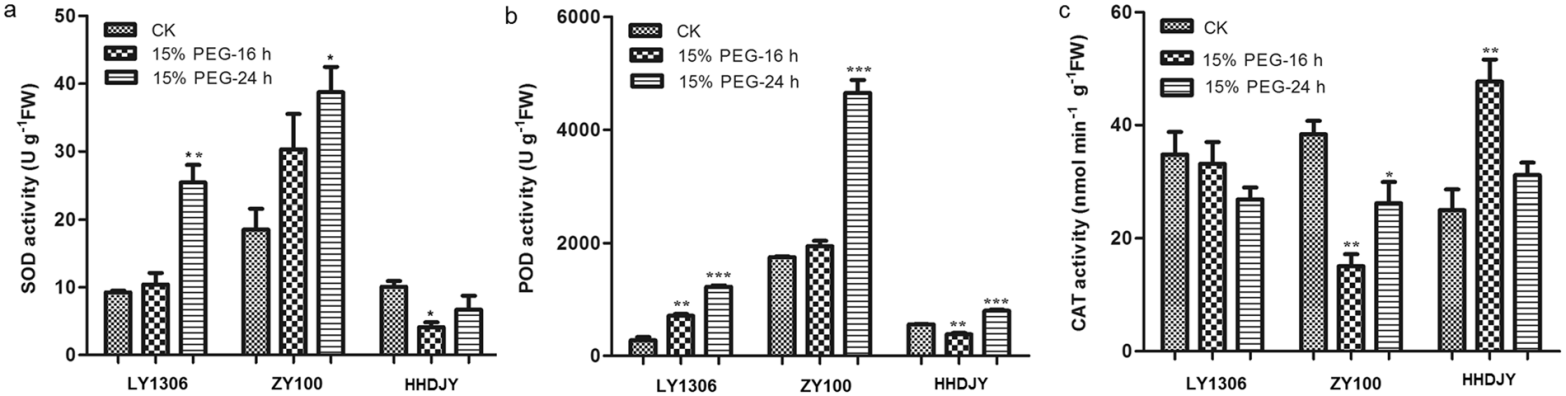
Changes in antioxidant enzyme activity of LY1306, ZhongYan 100 (ZY100) and Hong Hua Da Jin Yuan (HHDJY) tobacco strains after treatment with 15% PEG-6000 for 16 h and 24 h. (**a**) superoxide dismutase (SOD); (**b**) peroxide (POD) and (**c**) catalase (CAT). Results are expressed as the mean ± standard error from three replication experiments. *P < 0.05, **P < 0.01 and ***P < 0.001 compared with the corresponding control groups.

The activities of antioxidant enzymes, superoxide dismutase (SOD), peroxidase (POD) and catalase (CAT) after treatment with 15% PEG-6000 were investigated. LY1036 and ZY100 strains subjected to 15% PEG-6000 treatment exhibited significantly higher SOD activity (P < 0.05) than the controls after 24 h of treatment (175.12% for LY1306 and 109.31% for ZY100) (Fig. 2a). In addition, after 24 h of treatment, LY1036 and ZY100 exhibited significantly higher SOD activities (P < 0.01) than HHDJY. These results suggest that SOD activity plays an important role in drought resistance of LY1306.

Figure 2b presents changes in POD activities among the three tobacco strains treated with 15% PEG-6000. The POD activity of LY1036 showed a 155.65% increase (P < 0.01), that of ZY100 showed a 6.35% increase, whereas that of HHDJY showed a 31.07% decrease (P < 0.01) compared with those of the corresponding control groups after 16 h of treatment. Additionally, the POD activities of LY1036 and ZY100 were significantly higher (P < 0.01) than that of HHDJY. After 24 h, POD activities of all three tobacco strains significantly increased (P < 0.001) compared with those of the corresponding controls groups (321.03% for LY1306, 154.47% for ZY100 and 43.70% for HHDJY). Furthermore, the POD activities were higher in LY1036 and ZY100 than in HHDJY. Therefore, POD activity may be important for drought resistance in LY1306.

There was no significant difference in CAT activities among the three treatments groups in LY1306 (Fig. 2c). In contrast, in ZY100, the CAT activity in the 15% PEG-6000 treatment group was significantly lower (P < 0.05) than that in the control group. The CAT activity of HHDJY increased at first and then decreased. No significant difference was observed among the three tobacco strains after 24 h of 15% PEG-6000 treatment, suggesting that CAT activity does not influence drought resistance of LY1306.

### Effect of drought stress on MDA content of LY1306

**Figure 3:**
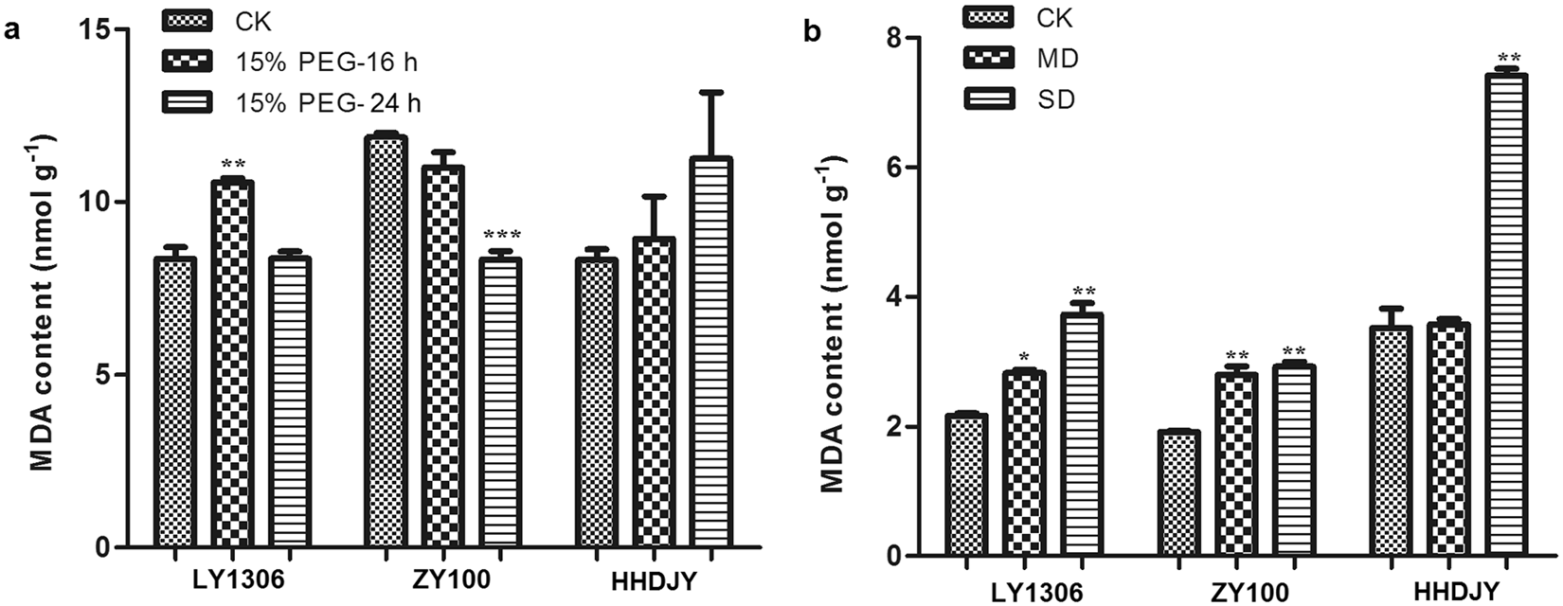
Changes in malondialdehyde (MDA) content of LY1306, ZhongYan 100 (ZY100) and Hong Hua Da Jin Yuan (HHDJY) tobacco strains after (**a**) being treated with 15% PEG-6000 for 16 h or 24 h and (**b**) withholding water supply. Results are expressed as the mean ± standard error from three replication experiments. *P < 0.05, **P < 0.01 and ***P < 0.001 compared with the corresponding control groups.

MDA content is an important indicator of the degree of peroxidation in plant cells. Variations in the MDA content were observed among the three tobacco strains after treatment with 15% PEG-6000 and after withholding water supply. After 16 h of 15% PEG-6000 treatment, the MDA content of LY1306 significantly increased (P < 0.01) and that of HHDJY slightly increased, whereas the MDA content of ZY100 decreased (Fig. 3a). After 24 h, the MDA content of LY1306 decreased to the same level as that exhibited by the corresponding MDA controls, whereas the MDA content of ZY100 significantly decreased (P < 0.001) compared with that of the corresponding MDA controls The MDA content of HHDJY continuously increased throughout the experiment. In general, after 24 h, HHDJY had the highest (P < 0.001) MDA content among the three strains. Under the water-deficit-induced drought stress, the MDA content of LY1306 and ZY100 significantly increased (P < 0.05 or 0.01) in MD group compared with control (Fig. 3b). Additionally, the MDA content in SD group was significantly higher than that in control in all of the three varities (P < 0.01). Moreover, the MDA content of HHDJY was significantly higher than that of LY1306 and ZY100 in MD and SD group (P < 0.01 or 0.001). These results suggest that MDA content is a key indicator of drought resistance in LY1306.

### Effect of drought stress on proline content of LY1306

**Figure 4:**
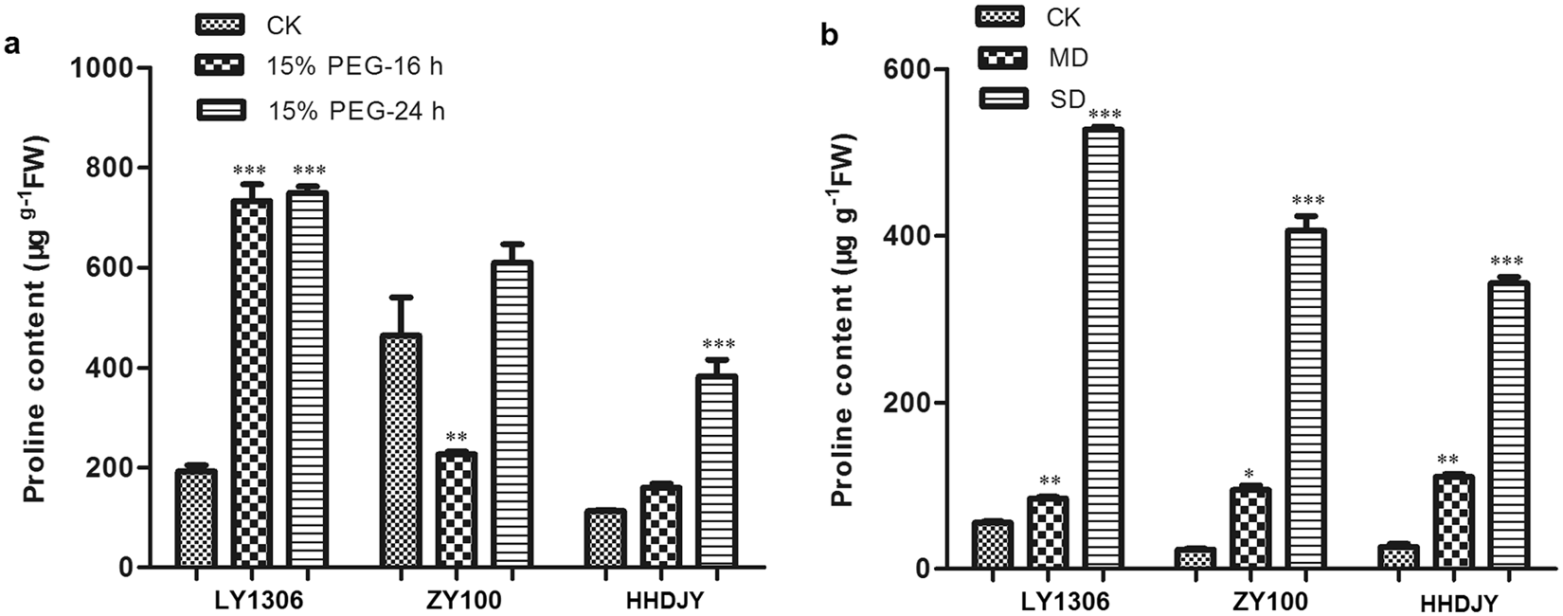
Changes in proline content of LY1306, ZhongYan 100 (ZY100) and Hong Hua Da Jin Yuan (HHDJY) tobacco strains after (**a**) being treated with 15% PEG-6000 for 16 h and 24 h and (**b**) withholding water supply. Results are expressed as the mean ± standard error from three replication experiments. *P < 0.05, **P < 0.01 and ***P < 0.001 compared with the corresponding control groups.

Proline plays an important role in plant osmoregulation, and the proline content changes under drought stress^16^. After 16 h of 15% PEG-6000 treatment, compared with the controls, the proline content increased by 280.78% in LY1306 (P < 0.001) and by 41.98% in HHDJY (P < 0.001), whereas it decreased by 51.27% in ZY100 (P < 0.01). After 24 h of treatment, the proline content of LY1306 and HHDJY continuously increased (P < 0.001) compared with those of the corresponding controls. Further, the proline content of ZY100 increased after 24 h of treatment (Fig. 4a). In addition, the proline content under drought stress was higher (P < 0.05) in LY1306 and ZY100 than that in HHDJY. When water supply was withheld, the proline content in the three tested tobacco strains increased (Fig. 4b). Furthermore, under SD, the proline contents of LY1306 and ZY100 were significantly higher (P < 0.05) than that of HHDJY. These results suggest that proline plays an important role in drought resistance of LY1306.

### Effect of drought stress on chloroplast ultrastructure in mesophyll cells

**Figure 5:**
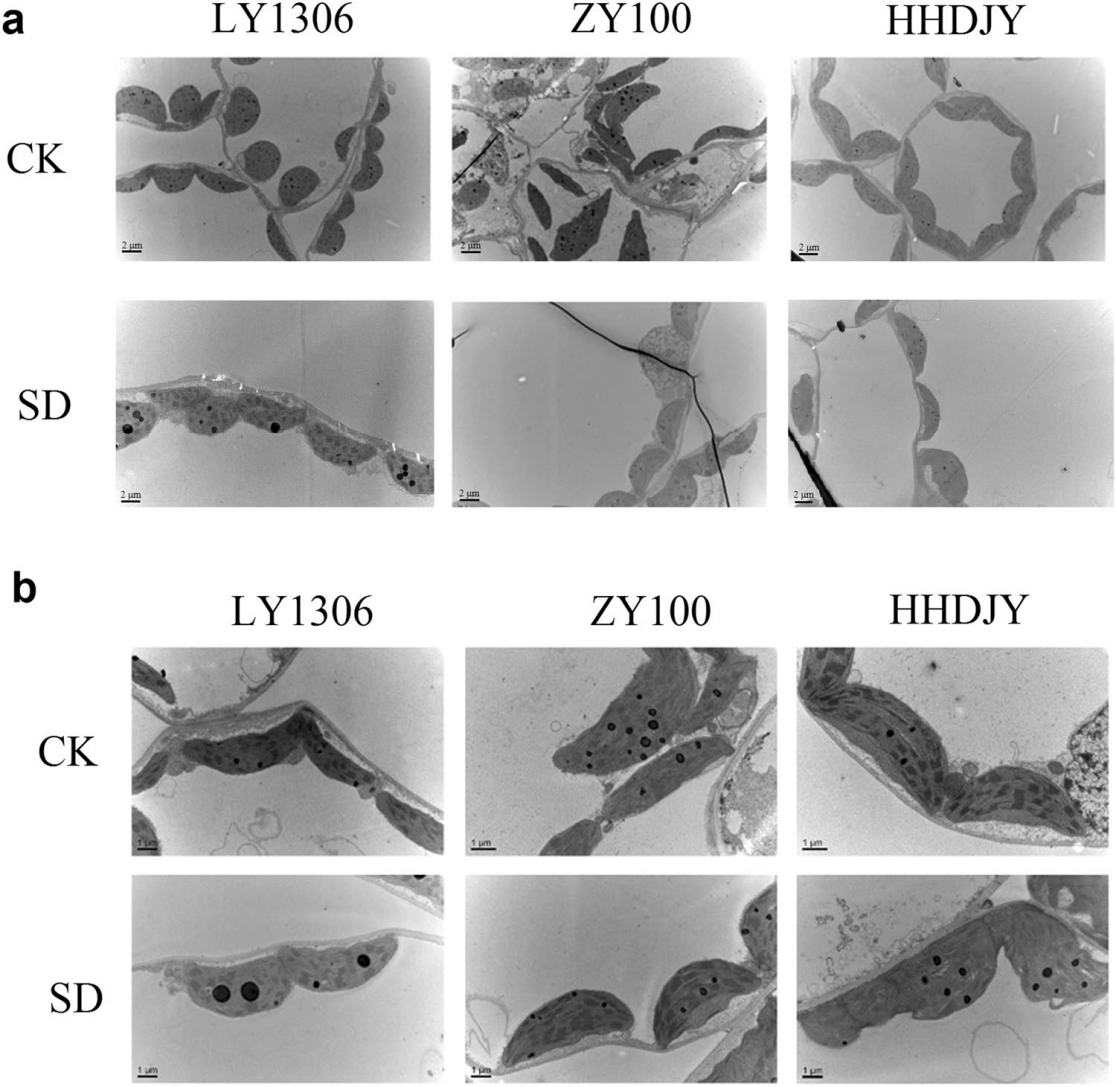
Chloroplast ultrastructure of mesophyll cells in LY1306, ZhongYan 100 (ZY100) and Hong Hua Da Jin Yuan (HHDJY) tobacco strains under severe drought (SD) conditions. (**a**) cellular structure (2 μm); (**b**) chloroplast ultrastructure (1 μm).

Under normal moisture conditions (CK), cell structure, chloroplasts morphology and ultrastructure among the three strains were similar. Under SD for 15 days, the number of chloroplasts decreased in all the three strains; however, the number of chloroplasts in LY1306 was more than that in HHDJY. Additionally, chloroplast morphology and thylakoid structure of LY1306 cells were normal. In HHDJY, chloroplasts showed shape change, and the lamellae of the thylakoids were disorganised (Fig. 5).

#### Sequence alignment

By mapping clean reads to the K326 tobacco reference genome, we obtained total map rates of each of the three tobacco strains (LY1306, 89.81%; ZY100, 91.62% and HHDJY, 91.18%); after mapping the clean reads to a K326 tobacco reference gene, total map rates of each of the three tobacco strains were 95.39%, 96.20% and 95.96%, respectively.

### Differentially expressed gene (DEG) analysis

In total, 3,066 DEGs including 1,449 up-regulated and 1,617 down-regulated genes, were identified between LY1306 and ZY100 strains. Additionally, 3126 DEGs including 1,660 up-regulated and 1,466 down-regulated DEGs were identified between LY1306 and HHDJY (Supplementary Table 1). Furthermore, 564 common up-regulated abd 434 common down-regulated DEGs were identified between LY1306 vs. ZY100 and LY1306 vs. HHDJY.

### Functional enrichment analyses

**Figure 6:**
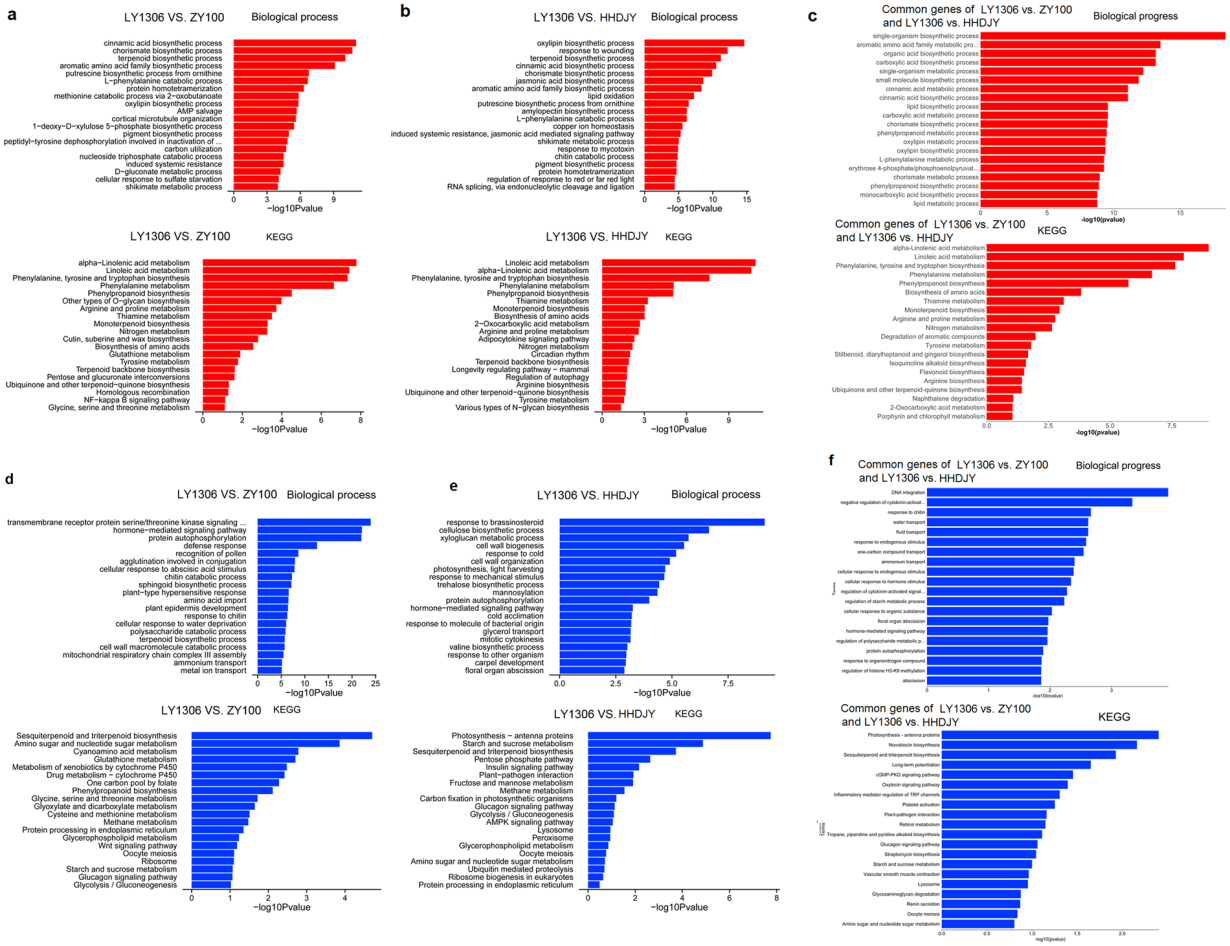
(**a**) Biological processes and KEGG pathways enriched by the up-regulated differentially expressed genes between LY1306 and ZhongYan 100 (ZY100) tobacco strains after being treated with 15% PEG-6000 for 16 h. (**b**) Biological processes and KEGG pathways enriched by the up-regulated differentially expressed genes between LY1306 and Hong Hua Da Jin Yuan (HHDJY) tobacco varieties after being treated with 15% PEG-6000 for 16 h. (**c**) Biological processes and KEGG pathways enriched by the common up-regulated differentially expressed genes of LY1306 vs. ZhongYan 100 (ZY100) and LY1306 vs. Hong Hua Da Jin Yuan (HHDJY). (**d**,**e** and **f**) Biological processes and KEGG pathways enriched by the down-regulated differentially expressed genes.

Gene ontology (GO) enrichment analysis showed that the up-regulated DEGs between LY1306 and ZY100 were mainly enriched with genes regulating biological processes associated with cinnamic acid, terpenoid and oxylipin biosyntheses. DEGs between LY1306 and HHDJY were mainly enriched with genes regulating biological processes, including oxylipin, terpenoid and jasmonic acid biosyntheses, wounding response and lipid oxidation. Kyoto Encyclopedia of Genes and Genomes (KEGG) pathway enrichment analysis revealed that the up-regulated DEGs between LY1306 and ZY100 were mainly enriched with genes regulating alpha-linolenic acid, linoleic acid, arginine and proline metabolisms. Additionally, these pathways were also enriched by DEGs between LY1306 and HHDJY (Fig. 6a and b). Furthermore, the common up-regulated DEGs were significantly enriched in GO terms associated with lipid and oxylipin biosyntheses and metabolisms, and pathways regulating alpha-linolenic acid, linoleic acid [such as mRNA_139072, mRNA_139073, and mRNA_139074 (all the three genes encoded linoleate 13S-lipoxygenase 2-1, chloroplastic-like, partial)], and arginine and proline metabolisms [such as mRNA_107515 (polyamine oxidase 1 isoform ×2), mRNA_109766 (putative amidase C869.01), and mRNA_142147 (S-adenosylmethionine decarboxylase proenzyme-like)] (Fig. 6c).

In addition to up-regulated DEGs, the down-regulated DEGs of LY1306 vs. ZY100 were enriched in GO terms related to metal ion transport, and ammonium transport, as well as pathways of Glycolysis/Gluconeogenesis, and Starch and sucrose metabolism. The down-regulated DEGs of LY1306 vs. HHDJY were enriched in GO terms about floral organ abscission, and carpel development, and pathways of Protein processing in endoplasmic reticulum and Ribosome biogenesis in eukaryotes. Moreover, the common down-regulated DEGs of LY1306 vs. ZY100 and LY1306 vs. HHDJY were significantly involved in GO functions related to DNA integration, and pathway of Photosynthesis-antenna proteins.

**Figure 7:**
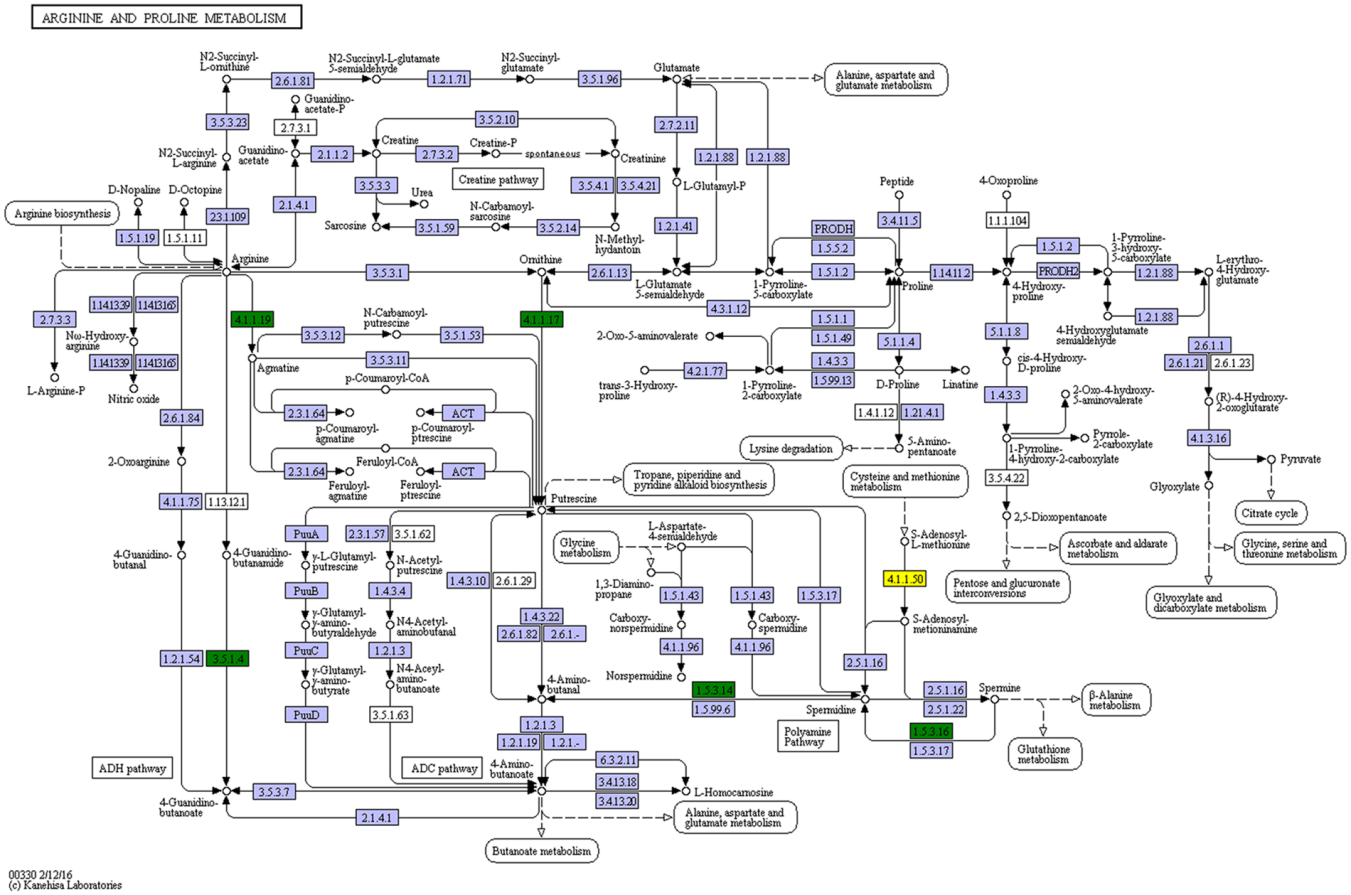
Regulatory network of arginine and proline metabolisms. Green boxes and yellow box represent the enriched DEGs (green includes up-regulated gene, yellow includes both up-regulated and down-regulated genes). The pathway map without coloring is the original version that is manually drawn by in-house software named KegSketch. White boxes are hyperlinked to KO, ENZYME, and REACTION entries in metabolic pathways, and to KO and GENES entries in non-metabolic pathways. Purple boxes are hyperlinked to KO entries that are selected from the original version.

Our biochemical experiments showed that proline plays an important role in drought resistance of LY1306, therefore, we displayed the pathway map of arginine and proline metabolisms (Fig. 7). In the KEGG map, 1.5.3.14 indicates mRNA_107515 (polyamine oxidase 1 isoform ×2); 3.5.1.4 indicates mRNA_109766 (putative amidase C869.01); 4.1.1.50 represents mRNA_142147 (S-adenosylmethionine decarboxylase proenzyme-like).

## Discussion

The present study investigated the drought resistance ability of a newly bred tobacco strain, LY1306, and further explored the potential mechanisms underlying the drought stress resistance using biochemical and transcriptomic approaches. Our results revealed that LY1306 exhibited better drought resistance than HHDJY. Our transcriptome sequencing analyses revealed thousands of DEGs between LY1306 and two control tobacco strains (ZY100 and HHDJY). These DEGs were significantly enriched in chemical compounds involved in biosynthetic and metabolic processes associated with GO terms and KEGG pathways. Many of these chemical compounds may be responsible for the drought resistance of LY1306.

Drought is a major environmental determinant of plant growth and productivity. Exposure to drought stress induces the generation of reactive oxygen species, which have a negative oxidative effect on cellular structures and metabolism^17^. Plants have developed some strategies to cope with this challenge. The antioxidant defence system is one of the drought stress defence mechanisms^18^. SOD converts O_2_^−^ into H_2_O_2_, POD reduces H_2_O_2_ to H_2_O based on the various substrates available as electron donors and CAT dismutates H_2_O_2_ into H_2_O and O_2_. Many studies have suggested that the activities of antioxidant enzymes are correlated with a plant’s drought resistance^19,20^. The activities of antioxidant enzymes are higher in stress-resistant species than in stress-sensitive species^21^. Our data revealed that SOD and POD activities of LY1306 and ZY100 were significantly higher than those of HHDJY under stress induced with 15% PEG-6000. Interestingly, there was no significant change in CAT activity of LY1306 under the various treatments.

MDA is a by-product of lipid peroxidation, which serves as suitable marker for membrane lipid peroxidation^22^, and the MDA content usually increases under stress damage^23^. In general, stress-sensitive tobacco strains have a higher MDA content and electrolyte leakage in response to environmental stress than stress-resistant strains^24^. In the present study, the MDA content gradually increased with increased drought stress in all the three strains. Interestingly, we also found that the MDA content of HHDJY was significantly higher than those of LY1306 and ZY100 under both MD and SD.

Proline can accumulate to high concentrations without damaging cellular macromolecules. Therefore, it acts as a compatible osmolyte. Importantly, proline provides protection against membrane damage and protein denaturation during severe drought stress^25^. Stress-mediated changes in free proline levels have been studied in various plant species, and several possible roles conferring stress resistance have been proposed^26^. Proline accumulation is a physiological response of plants subjected to drought stress^27^. Our results regarding proline accumulation were in agreement with the results of studies cited above. In the present study, proline accumulation in response to drought was observed in all the three strains. In particular, proline contents of LY1306 and ZY100 were significantly higher than that of HHDJY. Interestingly, our pathway enrichment analysis revealed that the DEGs between LY1306 and ZY100 and between LY1306 and HHDJY, such as mRNA_107515, mRNA_109766, and mRNA_142147, were up-regulated in LY1306 and significantly enriched in arginine and proline metabolisms. Therefore, proline accumulation in LY1306 under drought stress may be associated with mRNA_107515, mRNA_109766, and mRNA_142147 as well as arginine and proline metabolisms.

Plant resistance to drought involves not only morphological and biochemical adaptations but also responses at the genetic level^28^. In the present study, numerous DEGs were identified between LY1306 and ZY100, and between LY1306 and HHDJY. Some of these DEGs, such as mRNA_139072, mRNA_139073, and mRNA_139074 (all the three genes encoded linoleate 13S-lipoxygenase 2-1, chloroplastic-like, partial), are significantly involved in alpha-linolenic acid and linoleic acid metabolisms; these acids are important polyunsaturated fatty acids (PUFAs) associated with cell membrane lipids. Lipids are important membrane components and are severely affected by drought stress. Lipid composition changes maintain membrane integrity under water stress conditions^29^. In the biochemical experiments conducted in our study, the MDA content was hypothesised to be a key indicator of drought resistance of LY1306. MDA appears to be the most mutagenic product of lipid peroxidation^30^. In general, lipid peroxidation is described as a process under which oxidants attack lipids containing carbon–carbon double bond(s), particularly PUFAs^31^. Therefore, alpha-linolenic acid and linoleic acid metabolisms as well as their enriched genes (mRNA_139072, mRNA_139073 and mRNA_139074) may be associated with the MDA content in all the three tobacco strains.

Furthermore, our GO enrichment analysis found that these up-regulated DEGs were mainly enriched in genes that regulate biochemical processes associated with cinnamic acid, and jasmonic acid biosyntheses. Cinnamic acid identified from the root exudates of cucumber^32^ could inhibit plant growth by affecting ion uptake and hydraulic conductivity. Cinnamic acid has been reported to reduce lipid peroxidation and increase activities of antioxidant enzymes, such as SOD and POD, in drought-stressed cucumber leaves^33,34^. Jasmonic acid, belonging to a class of derived polyunsaturated fatty acid phytohormones, occurs ubiquitously in plants and induces a wide range of plant stress responses. In particular, jasmonic acid is effective in protecting plants from drought-induced oxidative damage^35^. A recent study has reported that jasmonic acid can modulate the antioxidant response mechanism of higher plants by tightly regulating biomembrane peroxidation^36^. Therefore, we speculated that the higher antioxidant enzymes activities in LY1306 in response to drought stress are associated with cinnamic acid and jasmonic acid biosyntheses.

Although our study investigated drought resistance mechanisms of LY1306 at the transcriptomic level and identified some DEGs and pathways that may play roles in drought resistance of LY1306, the relative expression of DEGs or the possible involvement of biochemical pathways in regulating the expression of DEGs was not further investigated. Therefore, our future study will focus on relevant research regarding these factors.

In conclusion, our study suggests that the LY1306 strain could significantly enhance the activities of antioxidant enzymes, increase the accumulation of proline and decrease the MDA content in plant cells as an adaptive response to drought stress. These drought resistance reactions may be associated with biochemical pathways of arginine, proline, alpha-linolenic acid and linoleic acid metabolisms, and cinnamic acid and jasmonic acid biosyntheses.

## Methods

### Plant materials and growth conditions

The LY1306 tobacco strain (provided by Luoyang tobacco company, Henan, China) and two control tobacco strains, ZhongYan 100 (ZY100) and Hong Hua Da Jin Yuan (HHDJY) (both provided by Henan Agricultural University, Henan, China) were used as biomodels in the present study. Tobacco seedlings were grown in a floating system for 46 days in a culture room (27 °C, 70% humidity and 16 h light).

### Drought treatment

For polyethylene glycol (PEG)-6000-induced osmotic stress, tobacco seedlings were transplanted into the perlite-based Murashige and Skoog (MS) medium (without sugar and agar) when they grew four leaves. The culture solution was changed every 3 days. After 23 days post-transplantation, either 25% or 15% PEG-6000 was added to the MS medium to induce osmotic stress for 5 h, 16 h or 24 h.

For water-deficit-induced drought stress, tobacco seedlings were transplanted into culture pots and grown in nutrient-amended soil. During the 35-day post-transplantation growth period, all seedlings were watered once a week (3 L at each time). After 40 days post-transplantation, the tobacco seedlings were randomly divided into CK, MD and SD groups. The control group seedlings were watered once every 5 days with 3 L of water; the MD group seedlings were watered once every 5 days with 1.5 L of water; and the SD group seedlings were not watered. All treatments lasted for 15 days. Then, watering was discontinued for all seedlings until visible wilting symptoms appeared in the drought treatment groups, which occurred within 20 days after watering ceased.

### Measurements

The changes in plant morphology, leaf water potential, chlorophyll content, antioxidant enzyme activity, MDA and proline contents and chloroplast ultrastructure in mesophyll cells of leaves in response to osmotic or drought stress were observed. For each measurement, three seedlings were randomly selected from each tobacco strain; leaves were used for all measurements. All the experiments were repeated thrice.The leaf water potential was detected with a pressure chamber (PMS Instruments, Corvallis, OR).

Chlorophyll contents were determined with alcohol. Briefly, the fresh leaves (0.2 g) were homogenized with 2-3 ml of 95% alcohol. Then 10 ml alcohol was added into the homogenate and ground until the tissue blanched. After 3 to 5 minutes’ standing, the extract liquid was filtered into brown volumetric flask (25 ml). The funnel, filter paper and mortar were washed with alcohol and liquid was added into the volumetric flask. Finally, alcohol was added to make 25 ml. The absorbance was measured at 665 nm, 649 nm and 470 nm, and chlorophyll concentrations were calculated as follows:

$${\rm{Chlorophylla}}:{\rm{Ca}}\,({\rm{mg}}/{\rm{l}})=13.95\times {{\rm{D}}}_{665}-6.88\times {{\rm{D}}}_{649}$$

$${\rm{Chlorophyllb}}:{\rm{Cb}}\,({\rm{mg}}/{\rm{l}})=24.96\times {{\rm{D}}}_{649}\,-\,7.32\times {{\rm{D}}}_{665}$$

$${\rm{Carotenoid}}=(1000\times {{\rm{D}}}_{470}-2.05\times {\rm{Ca}}\,-\,114.8\ast {\rm{Cb}})/245$$

Chlorophyll content was calculated as:

Chlorophyll content (mg/g) = chlorophyll concentration (mg/l) × volume of extract liquid/Dry weight of the sample (g)

SOD, POD and CAT activities were determined using SOD, POD and CAT microdetermination kits (Suzhou Comin Biotechnology Co., Ltd, Jiangsu, China), respectively. MDA and proline contents were also estimated using microdetermination kits (Suzhou Comin Biotechnology Co., Ltd).

The chloroplast ultrastructure of mesophyll cells was observed via transmission electron microscope detection^37^. Briefly, after sectioning, leaf samples (10 mm^2^ in size) were fixed in phosphate buffer (pH 7.2) containing 5% glutaraldehyde, followed with washing with 0.1 molL^−1^ phosphate buffer (pH 7.0) thrice. The washed samples were then fixed for 2 h in 1% osmic acid and prepared and washed three more times with the same phosphate buffer (pH 7.0). The washed samples were dehydrated with 30%, 50%, 70%, 90% and 100% ethanol and then placed into an embedding medium. The samples were then sectioned with a Leica EM UC7 ultramicrotome (Leica Microsystems GmbH, Wetzlar, Germany) and treated with uranyl acetate, followed by lead citrate. The samples were examined and photographed using an electron microscope (H-7650; Hitachi Ltd, Tokyo, Japan).

### Transcriptome sequencing

Transcriptome sequencing was performed on mRNAs obtained from the leaves of LY1306, ZY100 and HHDJY strains treated with 15% PEG-6000 for 16 h (Shanghai OE Biotech. Co., Ltd, Shanghai, China). Sequencing was performed on three leaves from each strain. Total RNAs were extracted from leaf tissue using mirVana™ miRNA ISOlation Kit (Ambion-1561), and DNA was digested with DNase. mRNAs were isolated from 50 μL total RNA using magnetic beads with an Oligo-dT tag. mRNAs were cleaved into short fragments, which were then used as templates to synthesise first-strand cDNA with random hexamer primers using the First Strand Synthesis Act D Mix and SuperScript II Reverse Transcriptase (Cat. No. 18064014; Invitrogen, CA, USA). Double-stranded cDNA was synthesised with the first-strand reaction using TruSeq Stranded mRNA LTSample Preparation Kit (Cat. No. RS-122-2101; Illumina, CA, USA). After purification of the double-stranded cDNA with the Agencourt AMPure XP system (Cat. No. A63881; Beckman Coulter, Brea, CA, USA), end repair, dA tailing and adaptor ligation were performed. Then, the end products were size-selected and enriched using PCR to create a cDNA library for transcriptome sequencing. Agilent 2100 Bioanaylzer (Agilent Technologies, CA, USA) was used to quantify and control the quality of the sample library. The paired-end libraries were then sequenced on the Illumina HiSeq^TM^ 2500 platform.

All data are available at the National Center for Biotechnology Information (NCBI) Sequence Read Archive database under the accession number SRP100649.

### Sequence alignment and gene expression analysis

The raw reads obtained from Illumina HiSeq^TM^ 2500 sequencing were processed by removing the low-quality reads, and reads with N. The obtained clean reads were aligned with the K326 tobacco reference genome (ftp://ftp.solgenomics.net/genomes/Nicotiana_tabacum/assembly/Ntab-K326_AWOJ-SS.fa.gz) and a reference gene (ftp://ftp.solgenomics.net/genomes/Nicotiana_tabacum/annotation/Ntab-K326_AWOJ-SS_K326.mrna.annot.fna) using TopHat software^38^ and Bowtie2^39^, respectively.

The gene expression level is directly reflected in the abundance of its transcript. Abundances were reported in expected fragments per kilobase of transcript per million fragments mapped (FPKM)^40^. FPKM is calculated based on the mapped transcript fragments, transcript length, and sequencing depth, which is the most commonly used method for estimating gene expression. In this study, we used reference genes to measure the abundance of each transcript according to sequence similarity alignment by using the software packages of Bowtie2^39^ (http://bowtie-bio.sourceforge.net/bowtie2/manual.shtml) and eXpress (http://www.rna-seqblog.com/express-a-tool-for-quantification-of-rna-seq-data/).

### DEGs and functional enrichment analyses

Based on the obtained gene expression levels, DEGs between LY1306 and ZY100 tobacco strains and between LY1306 and HHDJY tobacco strains were screened using the DESeq package^41^. (http://bioconductor.org/packages/release/bioc/html/DESeq.html) with an adjusted probability criterion of P ≤ 0.05.

The identified DEGs were subjected to the GO function and KEGG pathway enrichment analyses. The significance of enriched DEGs as per the GO terms or KEGG pathway maps was calculated according to their hypergeometric distributions. Any term or pathway that contained less than three genes was removed.

Additionally, due to the important role of proline in drought resistance, we downloaded the pathway map of arginine and proline metabolisms from KEGG PATHWAY database. In the KEGG map, green boxes and yellow box represent the enriched DEGs (green includes up-regulated gene, yellow includes both up-regulated and down-regulated genes). The pathway map without coloring is the original version that is manually drawn by in-house software named KegSketch. White boxes are hyperlinked to KO, ENZYME, and REACTION entries in metabolic pathways, and to KO and GENES entries in non-metabolic pathways. Purple boxes are hyperlinked to KO entries that are selected from the original version.

### Statistical analysis

Our results of biochemical experiments are presented as mean ± standard error. Statistical analyses were performed using SPSS 17.0. Differences were compared using analysis of variance (ANOVA) followed by least significant difference (LSD). P < 0.05 was considered as significant.

## Electronic supplementary material

supplementary table 1

## References

[1] Jaleel CA (2009). Drought stress in plants: a review on morphological characteristics and pigments composition. International Journal of Agriculture & Biology..

[2] Gao F (2015). Transcriptomic Analysis of Drought Stress Responses in Ammopiptanthus mongolicus Leaves Using the RNA-Seq Technique. Plos One..

[3] Shao HB (2008). Higher plant antioxidants and redox signaling under environmental stresses. Comptes Rendus Biologies..

[4] Kusaka M, Ohta M, Fujimura T (2005). Contribution of inorganic components to osmotic adjustment and leaf folding for drought tolerance in pearl millet. Physiologia Plantarum..

[5] Shinozaki K, Yamaguchi-Shinozaki K, Seki M (2003). Regulatory network of gene expression in the drought and cold stress responses. Current opinion in plant biology..

[6] Neumann PM (2008). Coping mechanisms for crop plants in drought-prone environments. Annals of Botany..

[7] Rushton PJ (2008). Tobacco transcription factors: novel insights into transcriptional regulation in the Solanaceae. Plant Physiology..

[8] Reed, T.D., Johnson, C., Semtner, P., Wilkinson, C. Flue-cured tobacco production guide. *Virginia Cooperative Extension, Petersburg*. (2012).

[9] Tassew Z, Chandravanshi BS (2015). Levels of nicotine in Ethiopian tobacco leaves. SpringerPlus..

[10] Organization, A. Projections of tobacco production, consumption and trade to the year 2010. Projections of Tobacco Production Consumption & Trade to the Year. (2003).

[11] Liu X, Baird WV (2003). Differential Expression of Genes Regulated in Response to Drought or Salinity Stress in Sunflower. Crop Science..

[12] Zhou JL (2007). Global genome expression analysis of rice in response to drought and high-salinity stresses in shoot, flag leaf, and panicle. Plant Molecular Biology..

[13] Kosová K, Vítámvás P, Prášil IT, Renaut J (2011). Plant proteome changes under abiotic stress–contribution of proteomics studies to understanding plant stress response. Journal of proteomics..

[14] Yuan-ying, J.X.-h.W. *et al* [Tobacco Research Institute of CAAS, Chinese Tobacco Genetics and Breeding Research (Northern) Center, Qingdao, 266101]; Development of a new flue-cured tobacco variety Zhongyan-100 (CF965) and its application evaluation [J]. *Acta Tabacaria Sinica*. **2** (2006).

[15] Zhang S-t (2007). Explore the feature of quality of Honghuadajinyuan variety. JOURNAL-HUNAN AGRICULTURAL UNIVERSITY..

[16] Yamada M (2005). Effects of free proline accumulation in petunias under drought stress. Journal of Experimental Botany..

[17] Bartels D, Sunkar R (2005). Drought and salt tolerance in plants. Critical reviews in plant sciences..

[18] Foyer CH, Noctor G (2005). Oxidant and antioxidant signalling in plants: a re‐evaluation of the concept of oxidative stress in a physiological context. Plant, Cell & Environment..

[19] Demiral T, Türkan I (2005). Comparative lipid peroxidation, antioxidant defense systems and proline content in roots of two rice cultivars differing in salt tolerance. Environmental and Experimental Botany..

[20] Türkan İ, Bor M, Özdemir F, Koca H (2005). Differential responses of lipid peroxidation and antioxidants in the leaves of drought-tolerant P. acutifolius Gray and drought-sensitive P. vulgaris L. subjected to polyethylene glycol mediated water stress. Plant Science..

[21] Bor M, Özdemir F, Türkan I (2003). The effect of salt stress on lipid peroxidation and antioxidants in leaves of sugar beet Beta vulgaris L. and wild beet Beta maritima L. Plant Science..

[22] Qiu Z (2012). He-Ne laser pretreatment protects wheat seedlings against cadmium-induced oxidative stress. Ecotoxicology & Environmental Safety..

[23] GUO Z-q (2008). The Effects of Chilling-resistant Agents on Corn Growth, Physiological and Biochemical Changes in Low Temperature Stress. Journal of Maize Sciences..

[24] Dionisio-Sese ML, Tobita S (1998). Antioxidant responses of rice seedlings to salinity stress. Plant Science..

[25] Ain-Lhout F (2001). Comparison of proline accumulation in two Mediterranean shrubs subjected to natural and experimental water deficit. Plant and soil..

[26] Koca H, Bor M, Özdemir F, Türkan I (2007). The effect of salt stress on lipid peroxidation, antioxidative enzymes and proline content of sesame cultivars. Environmental and Experimental Botany..

[27] Planchet, E. *et al*. Abscisic acid-induced nitric oxide and proline accumulation in independent pathways under water-deficit stress during seedling establishment in Medicago truncatula. *Journal of Experimental Botany*. eru088 (2014). 10.1093/jxb/eru088 24604737

[28] Levitt, J. *Responses of plants to environmental stresses*, Academic Press (1980).

[29] Paula FMD (1990). Effects of water stress on the molecular species composition of polar lipids from Vigna unguiculata L. leaves. Plant Science..

[30] Esterbauer H, Eckl P, Ortner A (1990). Possible mutagens derived from lipids and lipid precursors. Mutation Research/Reviews in Genetic Toxicology..

[31] Ayala, A., Muñoz, M.F., Argüelles, S. Lipid peroxidation: production, metabolism, and signaling mechanisms of malondialdehyde and 4-hydroxy-2-nonenal. *Oxidative medicine and cellular longevity*. **2014** (2014). 10.1155/2014/360438 PMC4066722 24999379

[32] Yu JQ, Matsui Y (1994). Phytotoxic substances in root exudates of cucumber (Cucumis sativus L.). Journal of Chemical Ecology..

[33] Sun W-J (2012). Exogenous cinnamic acid regulates antioxidant enzyme activity and reduces lipid peroxidation in drought-stressed cucumber leaves. Acta Physiologiae Plantarum..

[34] Yu JQ, Ye SF, Zhang MF, Hu WH (2003). Effects of root exudates and aqueous root extracts of cucumber (Cucumis sativus) and allelochemicals, on photosynthesis and antioxidant enzymes in cucumber. Biochemical Systematics and Ecology..

[35] Mahmood M, Bidabadi SS, Ghobadi C, Gray DJ (2012). Effect of methyl jasmonate treatments on alleviation of polyethylene glycol -mediated water stress in banana (Musa acuminata cv. ‘Berangan’, AAA) shoot tip cultures. Plant Growth Regulation..

[36] Sirhindi G (2015). Modulatory role of jasmonic acid on photosynthetic pigments, antioxidants and stress markers of Glycine max L. under nickel stress. Physiology and Molecular Biology of Plants..

[37] XiaoYing L (2011). Regulation of chloroplast ultrastructure, cross-section anatomy of leaves, and morphology of stomata of cherry tomato by different light irradiations of light-emitting diodes. HortScience..

[38] Trapnell C, Pachter L, Salzberg SL (2009). TopHat: discovering splice junctions with RNA-Seq. Bioinformatics..

[39] Langmead B, Salzberg SL (2012). Fast gapped-read alignment with Bowtie 2. Nature methods..

[40] Trapnell C (2010). Transcript assembly and quantification by RNA-Seq reveals unannotated transcripts and isoform switching during cell differentiation. Nature biotechnology..

[41] Anders, S. & Huber, W. Differential expression of RNA-Seq data at the gene level – the DESeq package. *Embl* (2013).

